# Within-Niche Constant Selection of *Drosophila* Inversions?

**DOI:** 10.6064/2012/140859

**Published:** 2012-07-03

**Authors:** José M. Álvarez-Castro, Gonzalo Alvarez

**Affiliations:** Department of Genetics, University of Santiago de Compostela, Avenida Carvalho Calero s/n, 27002 Lugo, Galiza, Spain

## Abstract

Extensive and fruitful work is being devoted for more than 70 years to elucidate the fine points of the maintenance of inversion polymorphisms of the genus *Drosophila*. Recent studies have resumed selection in heterogeneous environments (or niches) as a major underlying mechanism for these balanced polymorphisms. In those studies, constant selection within niches is assumed throughout although this assumption is since long known not to hold. In the present communication it is sustained that the results in those studies are robust in the face of this fact. To that end, this communication deals with a particular long-lasting question within this topic—whether the minimal model of constant viability selection (MCV, assuming frequency-, sex-, and stage-independent adaptive values) suffices to reproduce the trajectories of frequencies of Drosophila chromosomal arrangements observed in experimental populations along generations under homogeneous environments. Fitness estimates are here obtained from published trajectories of frequencies using a maximum likelihood approach, and relevant literature is revised in the light of these new analyses, pointing to an affirmative answer to that question.

## 1. Introduction

Dobzhansky's fundamental finding that natural selection acts on polymorphic chromosomal inversions of *Drosophila* flies [1–3] was path-breaking in evolutionary biology and bestowed plenty of delightful work upon geneticists for years to come [4]. In particular, it triggered a long-term line of work to elucidate the particular, strong mechanism(s) of selection underlying the inversion polymorphisms that were maintained both under natural and (often) under experimental conditions [5, 6]. The first hypothesis to test was the heterozygote types (heterokaryotypes) having higher adaptive values than the homozygote types (homokaryotypes) assuming the model of constant viability (MCV), which was referred to as heterosis [7, 8]. The plausibility of this hypothesis did not only come from its simplicity, but also because no discrepancies were initially found between the predicted trajectories assuming heterosis and the observed data [5, 6, 8]. Subsequently, more thorough assessments were designed in order to definitely reveal the role of heterosis in the maintenance of the *Drosophila* inversion polymorphisms.

The least-squares and goodness-of-fit-based methods to estimate constant adaptive values from experimental runs [6, 9, 10] were replaced by more convenient maximum likelihood (ML) approaches [11, 12]. However, when applying these methods to real data, the results were not conclusive. For some experiments, the trajectories predicted using the estimated adaptive values would fit the data well, whereas statistically significant departures between the observed and the predicted trajectories would be found for others [12–14]. Further ML methods were then designed to account for more realistic selection regimes involving stage- and sex-dependent adaptive values [15, 16]. However, the data requirements increase with the number of parameters, and the information content the observed trajectories of frequencies can bear is limited. Consequently, Prout [17, 18] proposed that this puzzle would have to be broken up into more accessible pieces, which turned researchers in this field to estimate separate fitness components in competition experiments (reviewed in [19–21]). This line of research revealed the maintenance of *Drosophila* inversion polymorphisms not to be ruled simply by heterosis. In fact, selection has been shown to take capricious enough appearances that can simultaneously be frequency-, sex-, and stage-dependent, as rare male advantage [22, 23].

The integrative approach proposed by Prout [15, 17, 18, 24] consists in modelling the trajectories of frequency of *Drosophila* inversions along generations using the fitness-component estimates obtained in competition experiments. This strategy has recently shown a more than reasonably good fit of predicted-to-observed trajectories, using from the last ones only their starting points [25, 26]. However, this positive result is not sufficient for completely understanding the balanced inversion polymorphisms of *Drosophila's* natural populations, with individuals migrating among niches with different selection pressures [27–30]. In fact, *Drosophila* inversions being affected by selection in heterogeneous environments have been originally pointed out by Dobzhansky [2] as the cause of the altitudinal clines of their frequencies. Kirkpatrick and Barton [31] have more recently inspected the ecological and genetic mechanisms underlying the evolution of these clines and emphasized the potential of inversions for the adaptation of populations to local environments (i.e., niches). More to the point, Schaeffer [32] has recently estimated adaptive values of inversions for the six niches identified for *Drosophila pseudoobscura* in the southwestern USA, using a model of selection-migration balance.

In order to address the complexity of niche-dependent selection in those recent studies, the MCV has been used to describe selection within niches (or localities). However, *Drosophila* inversions are since long known to be affected by more complex modes of selection—as pointed out above. Consequently, the question of whether the reliability of those results is spoiled by the assumption of within-niche constant selection is here addressed. Dobzhansky's unresolved concern on whether the MCV fits the changes in frequency of *Drosophila* inversions [6] is thus revisited. More precisely, is the MCV (in spite of not being causative) appropriate to accurately reproduce the changes in frequency of *Drosophila* chromosomal arrangements along generations under constant environments? Previous studies on this subject are here reviewed in the light of new analyses of published data.

## 2. Methods

Estimates of stage-, sex-, and frequency-independent adaptive values and initial frequencies of three-allele karyotypic classes are here obtained from experimental populations of *Drosophila subobscura *[33] using an implementation of DuMouchel and Anderson's [12] unconditional ML method. The de Frutos' [33] datasets are of the same kind as the ones generated for using this ML method [12–14, 34, 35]—to this regard, it is in particular important to note that the experimental population sizes are large relative to the sample sizes so that the sample procedure does not strongly affect the population frequencies of subsequent generations [36]. The required implementation consists in estimating the initial frequencies, due to the experimental populations having been started from wild individuals with unknown frequencies [33]. This implementation was reported to have already been applied to other datasets [13, 14], although not yet described.

For a *k*-allele genetic system, the MCV expresses the gene frequencies of zygotes of generation (*t* + 1), *p*_*i*_(*t* + 1),  *i* = 1,…, *k*, in terms of the frequencies at the previous generation and the adaptive values *p*_*i*_(*t*), *ω*_*ij*_, *i*, *j* = 1,…, *k*, respectively, using the following recurrence equations:

(1) pi(t+1)=Fi(t)D(t), i=1,…,k,

where  *F*_*i*_(*t*) = *p*_*i*_(*t*)∑_*j*=1_^*k*^ *p*_*j*_(*t*)*ω*_*ij*_, *i* = 1,…, *k*, and *D*(*t*) = ∑_*l*,*m*=1_^*k*^ *p*_*l*_(*t*)*p*_*m*_(*t*)*ω*_*lm*_. The vector of independent parameters to estimate is **E** = (*p*_1_(0),…,*p*_*k*−1_(0),*ω*_11_,…,*ω*_*kk*−1_)^T^, where the superindex T stands for transpose. The remaining frequency, *p*_*k*_(0), is defined by all frequencies having to sum up to one, and the remaining adaptive value, *ω*_*kk*_, is defined by ∑_*ij*∈*V*_ *ω*_*ij*_ = *k*(*k* + 1)/2 where *V* is the set of the *k*(*k* + 1)/2 adaptive values.

The data are the observed gene frequencies in zygotes of subsequent, not necessarily consecutive, generations *x*_*i*_(*t*), *i* = 1,…, *k*, *t* ∈ *X*, and the number of genes sampled for each generation, *n*(*t*), *t* ∈ *X*, where *X* is the set of all observed generations. Thus, the likelihood function is ∏_*t*∈*X*_∏_*i*=1_^*k*^ *p*_*i*_(*t*)^*x*_*i*_(*t*)^. Hence, its logarithm is

(2) $$\begin{array}{c} L=c+\sum_{t\in X}\;\sum_{i=1}^{k}x_{i}\left(t\right)\log p_{i}\left(t\right), \end{array}$$

where *c* is a constant.

The ML estimate of the vector **E** can be computed iteratively from an attempting initial value **E** ^(*u*)^, using Newton's iteration algorithm **E** ^(*u*+1)^ = **E** ^(*u*)^ + **I** ^−1^ · **S** [37, 38], where the likelihood vector is **S** = (∂*L*/∂*ε*)_*ε*∈**E**_ = (∑_*t*∈*X*_∑_*i*=1_^*k*^(*x*_*i*_(*t*)/*p*_*i*_(*t*))(∂*p*_*i*_(*t*)/∂*ε*))_*ε*∈**E**_, using (2), and the information matrix is **I** = (∑_*t*∈*X*_ *n*(*t*)∑_*i*=1_^*k*^[((∂*p*_*i*_(*t*)/∂*δ*)(∂*p*_*i*_(*t*)/∂*ε*))/*p*_*i*_(*t*)])_*δ*,*ε*∈**E**_. In these expressions, the derivatives of the gene frequencies along generations with respect to the parameters to estimate can be computed recursively, from (1), as

(3) ∂pi(t+1)∂ε=∂Fi(t)∂ε1D(t)−Fi(t)[D(t)]2∂D(t)∂ε, ε∈E,             i=1,…,k,

 where

(4) ∂D(t)∂ε=∑i=1k∂Fi(t)∂ε, ε∈E,∂Fi(t)∂pr(0)=∑j=1k[ωij(pi(t)∂pj(t)∂pr(0)+pj(t)∂pi(t)∂pr(0))],            r=1,…,k−1,∂Fi(t)∂ωlm=∑j=1k[pi(t)pj(t)∂ωij∂ωlm   +ωij(pi(t)∂pj(t)∂ωlm+pj(t)∂pi(t)∂ωlm)],            lm∈V∖{kk}.

The recursive process is initiated using that the frequencies at time *t* = 0 are independent of the adaptive values and that ∂*p*_*i*_(0)/∂*p*_*r*_(0) equals 1, − 1, and 0, when *i* = *r*, *i* = *k*, and otherwise, respectively.

## 3. Results

**Figure 1:**
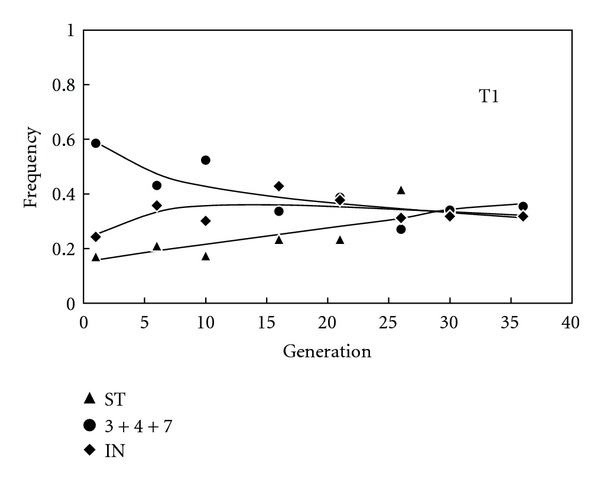
Observed frequencies (symbols) of arrangements of the *O* chromosome along generations of experimental population T1 of *Drosophila subobscura* [33] and predicted trajectories (lines) using ML estimates of frequency-, sex-, and stage-independent adaptive values and initial frequencies from the observed frequencies.

**Figure 2:**
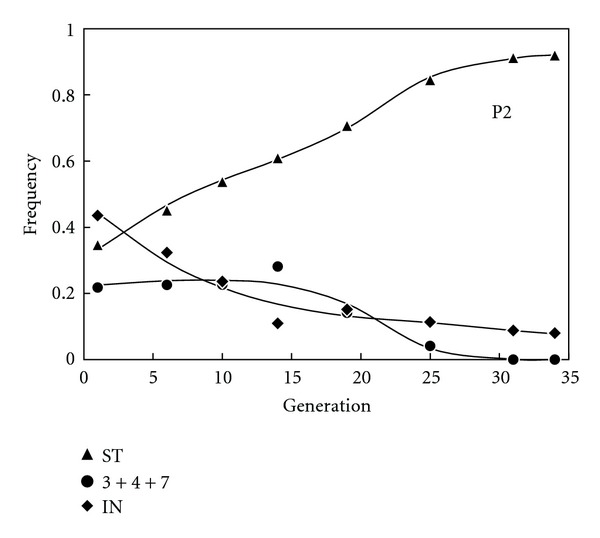
Observed frequencies and predicted trajectories of population P2 (same specifications as in legend of Figure 1).

The ML method described above succeeded to converge to a vector of positive estimates for only three out of the ten populations of *Drosophila subobscura* sampled along several generations by de Frutos [33]—the ones labelled as H2, T1, and P2 (Figures 1 and 2). These estimates are shown in Table 1 together with the results of a statistical test assessing the goodness of fit between the observed trajectories of frequencies and the ones predicted using the estimates. For populations H2 and T1, only a few of the multiple scrutinizing starting values have led to local convergence of the ML method, which reflects that the information content of the data is not optimal and the estimates are therefore not robust. In particular, H2 has a lower number of generations sampled than the other two populations and T1 has lower efforts of per generation samples than P2 (cf. sample sizes in [33]) and was funded at frequencies closer to the equilibrium (cf. Figures 1 and 2). In any event, the fact that populations associated to weak estimates (with only local convergence of the ML method) display statistically significant departures between the observed trajectories and the ones predicted by the estimates, using a goodness-of-fit test (Table 1), does not prove that the model cannot fit more informative data.

The key to address this problem is thus the performance the predictions show at populations for which global convergence to meaningful adaptive values evidences the good quality of the data. In point of fact, such data is scarce in the literature. Fortunately, however, this actually is the case for population P2 (Figure 2), where global convergence of the ML method described above has led to robust estimates of selective values (Table 1). In fact, the predicted trajectories for population P2 (Figure 2) seem to provide the best possible approximation to the data—the only mismatch attracting visual attention happens at generation 14, at which the least sampling effort for this population has been made [33]. This visual appreciation is in accordance with the results of the statistical tests. It is noteworthy that sample size is higher for P2 than for T1 [33] and that the larger the sample sizes, the higher the power to detect discrepancies between the predicted and the observed trajectories by the goodness-of-fit test [39]. In spite of that, no significant discrepancies occur for population P2 (Table 1). Therefore, these results clearly point to the estimation procedure to generate predicted trajectories that fit the data extremely well—whenever applied to datasets that are informative enough to provide robust estimates.

These results enable us to provide a coherent interpretation of the results reported in the literature for the inversion polymorphism of *Drosophila pseudoobscura* [12–14, 34, 35]. Indeed, reinterpreting those works has been a major motivation for us to use the same methodology. DuMouchel and Anderson [12] found no discrepancies between predicted (under the MCV) and observed trajectories in diallelic populations, but statistically significant discrepancies in multiallelic populations (in which similar sampling efforts were made in spite of the increasing number of parameters to estimate). Accordingly, statistically significant discrepancies between predicted and observed data were found by Watanabe et al. [13] in a highly parameterized genetic system (four-allele populations in which also the initial frequencies had to be estimated), whereas Anderson et al. [14] obtained good fit using lesser parameterized models (triallelic populations) and taking special care in sampling a considerable number of generations ahead of the equilibrium.

Overall, a comprehensive view of the estimation of selection (with the MCV) of *Drosophila* inversions from changes in frequency along generations can be summarized in two points. On the one hand, the statistical discrepancies found at populations between observed trajectories and the ones predicted with non robust estimates reflect that the amount of information content of many datasets is not in accordance with the number of parameters to estimate. On the other hand, the systematic fit of trajectories predicted by a reasonably small amount of estimates obtained from reasonably high-quality data (ensuing global convergence of the estimation procedure to meaningful values) proves that the constant (stage-, sex-, and frequency-independent) model of selection suffices to describe the observed trajectories of inversion frequencies within niches.

## 4. Discussion

Several recent studies have dealt with intraniche constant selection of *Drosophila* inversions as a black-box model to investigate how selection works across niches [31, 32]. This practice is in accordance with Prout's [17, 18] proposal of addressing, in turn, separate aspects of this problem. Schaeffer [32], in particular, provides evidence for selection in heterogeneous environments to be a crucial mechanism in the maintenance of inversion polymorphisms in *Drosophila* populations of the southwestern USA. He shows that models assuming constant adaptive values that do not display heterosis within niches can fit data on balanced polymorphisms in natural populations of *Drosophila pseudoobscura*. The adaptive values obtained under the different environments are then used to reproduce the equilibrium frequencies through recursions using the MCV with selection-migration balance, assuming different migration rates and migration schemes.

The motivation for the present communication is that the results just mentioned can be questioned by arguing that, within environments, adaptive values of *Drosophila* inversions are known not to be constant at all but stage-, sex-, and frequency-dependent, as explained in the Introduction section. Interestingly, the reasoning behind these results does not exactly rely on the MCV entailing the true mechanism of selection within the different niches. To be precise, Schaeffer's [32] results rely instead on the MCV being able to accurately reproduce the changes in inversion frequencies along generations of flies within each niche. Therefore, the apparent contradiction coming from using the MCV as a simplifying assumption vanishes under the outcome of this communication—the MCV, although non causative, suffices to reproduce the changes in frequency of *Drosophila* inversions that are due to selection within niches. Thus, a critical step has here been worked out that is needed to sustain previous results that have been published concerning the maintenance of *Drosophila* inversion polymorphisms.

It is not astonishing that the question of whether the MCV would fit *Drosophila* inversions frequencies lasted long. Indeed, when this subject was first addressed, the MCV was still regarded as a potential causative mechanism—instead of as a black-box model—for *Drosophila* inversion polymorphisms and heterosis comprised an appealing explanation to *Drosophila* balanced polymorphisms, as explained in the Introduction section. This fact must have initially encouraged researchers to address the estimation of adaptive values in all kinds of populations (including rather complex ones) and to progressively abandon this line of work as the evidences of complex modes of selection of inversions became stronger. As a consequence—and despite several indications advised for the design of experiments to estimate constant-selection parameters from trajectories of frequency (see, e.g., [12])—the *Drosophila* literature does not in the end provide many datasets that can lead to robust estimates. Rather, the experimental datasets often involve three or more alleles and entail the initial frequencies as parameters to estimate.

In the present communication it was actually necessary to reduce the complexity of the original data towards a triallelic system by pooling the less frequent arrangements into the category *O*_IN_. This is one of the factors putatively precluding most of the populations of de Frutos [33] to lead to global convergence of the ML method. On the other hand, the fitness estimate obtained for population P2 predicting the fixation of one arrangement, *O*_ST_ (Table 1), is an occasional fact (several inversions are often maintained in experimental populations, see, e.g., [21]) that actually facilitates the convergence of the estimation procedure—since it facilitates that a higher number of generations involving changes in frequency occur before the equilibrium is attained (cf. Figures 1 and 2). Incidentally, the estimates obtained by the ML method are consistent with the output of all populations since they correctly predict whether the multiallelic polymorphism would be maintained or not (see the equilibrium frequencies predicted from the estimates of adaptive values in Table 1), which reinforces our main conclusion—the MCV can be used to obtain adaptive values that approximate well the trajectories of frequencies of *Drosophila* inversions within niches, as long as there is enough data available for the ML method to provide robust estimates.

In any case, it must be recalled that the good fit of the MCV to the inversion frequencies cannot be argued to endorse heterosis as the causative factor of the maintenance of the *Drosophila* inversion polymorphisms. As pointed out above, numerous studies found complex modes of selection to occur in the maintenance of *Drosophila* inversion polymorphisms reviewed in [19–21]. Furthermore, the stage-, sex-, and frequency-dependent fitness estimates obtained in competition experiments of *Drosophila pseudoobscura* have been successfully used to replicate the trajectories of frequencies of experimental populations along generations [25], which supports those multifaceted fitness estimates—instead of the minimal MCV with heterosis—as the selection mechanisms underlying the maintenance of inversion polymorphisms in experimental populations. Similarly, it cannot be argued that selection in heterogeneous environments is the only force maintaining the polymorphisms, since other balancing forces (different from heterosis) are known to act within niches.

Dobzhansky's fundamental finding that *Drosophila* inversions are affected by strong selective forces in natural populations via seasonality [1] and altitudinal clines [2] gave rise to extensive, fruitful research in evolutionary biology during Dobzhansky's life and shortly after his passing reviewed in [21]. This topic keeps on improving nowadays our insight in new scientific challenges, as shown, for instance, through the assessment of global climate change by shifts of latitudinal clines of *Drosophila* inversion polymorphisms [40] and through the understanding of speciation mechanisms that may underlie the origin of humans [41]. On the whole (although dealing here only with what is related to the maintenance of *Drosophila* inversion polymorphisms), Dobzhanky's bequest keeps on bestowing plenty of motivating challenges upon geneticists for times to come.

## Figures and Tables

**Table 1:** ML estimates of frequency-, sex-, and stage-independent adaptive values, *ω*_*ij*_, and initial frequencies, *p*_*i*_(1), from observed frequencies of arrangements of the *O* chromosome along generations of experimental populations of *Drosophila subobscura* [33], goodness of fit, *χ* ^2^, and degrees of freedom, df, to test the adequacy of the selection model to the data and equilibrium frequencies, ${\hat{p}}_{i}$, predicted by the estimates.

Population	Arrangement^(a)^	*p*_*i*_(1) ± *σ*_*pi*(1)_	*ω*_1*j*_ ± *σ*_*ω*1*j*_	*ω*_2*j*_ ± *σ*_*ω*2*j*_	*ω*_3*j*_ ± *σ*_*ω*3*j*_	*χ* ^2^	df	${\hat{p}}_{i}$
	*O*_ST_	0.040 ± 0.011	0.936 ± 0.456	1.450 ± 1.672	1.083 ± 0.206	17.373^∗∗∗^	3	0.424
H2	*O*_3+4+7_	0.735 ± 0.026		0.367 ± 3.462	1.707 ± 4.584			0.338
	*O*_IN_	0.225 ± 0.024			0.456 ± 3.108			0.238

	*O*_ST_	0.158 ± 0.022	1.030 ± 0.115	1.045 ± 0.083	1.107 ± 0.259	26.105^∗∗∗^	7	0.596
T1	*O*_3+4+7_	0.590 ± 0.032		0.812 ± 0.078	1.240 ± 0.225			0.171
	*O*_IN_	0.252 ± 0.029			0.766 ± 0.308			0.233

	*O*_ST_	0.333 ± 0.024	1.069 ± 0.041	0.613 ± 0.103	1.045 ± 0.043	8.072	7	1
P2	*O*_3+4+7_	0.225 ± 0.019		1.963 ± 0.286	0.551 ± 0.219			0
	*O*_IN_	0.443 ± 0.029			0.759 ± 0.125			0

^(a)^The less frequent arrangements are pulled together into the category *O*_IN_, which is dominated by arrangements *O*_3+4_ or *O*_7_ [33].

***P < 0.001.

## Abbreviations

MCV: Model of constant viability
ML: Maximum likelihood.
